# COVID‐19 lockdown consequences on body mass index and perceived fragility related to physical activity: A worldwide cohort study

**DOI:** 10.1111/hex.13282

**Published:** 2021-06-09

**Authors:** Constanta Urzeala, Martine Duclos, Ukadike Chris Ugbolue, Aura Bota, Mickael Berthon, Keri Kulik, David Thivel, Reza Bagheri, Yaodong Gu, Julien S. Baker, Nicolas Andant, Bruno Pereira, Karine Rouffiac, The Covistress Network, Stéphanie Mestres, Cécile Miele, Valentin Navel, Lénise Parreira, Yves Boirie, Jean‐Baptiste Bouillon‐Minois, Maria Livia Fantini, Jeannot Schmidt, Stéphanie Tubert‐Jeannin, Pierre Chausse, Michael Dambrun, Sylvie Droit‐Volet, Julien Guegan, Serge Guimond, Laurie Mondillon, Armelle Nugier, Pascal Huguet, Samuel Dewavrin, Fouad Marhar, Geraldine Naughton, Amanda Benson, Claus Lamm, Vicky Drapeau, Raimundo Avilés Dorlhiac, Benjamin Bustos, Haifeng Zhang, Peter Dieckmann, Binh Quach, Yanping Duan, Gemma Gao, Wendy Y J Huang, Ka Lai Kelly Lau, Chun‐Qing Zhang, Jiao Jiao, Kuan‐chou Chen, Hijrah Nasir, Perluigi Cocco, Rosamaria Lecca, Monica Puligheddu, Michela Figorilli, Morteza Charkhabi, Daniela Pfabigan, Peter Dieckmann, Samuel Antunes, David Neto, Pedro Almeida, Maria João Gouveia, Pedro Quinteiro, Benoit Dubuis, Juliette Lemaignen, Andy Liu, Foued Saadaoui, Maëlys Clinchamps, Frédéric Dutheil

**Affiliations:** ^1^ Sports and Motor Performance Department Faculty of Physical Education and Sports National University of Physical Education and Sports from Bucharest Bucharest Romania; ^2^ Department of Sport Medicine and Functional Exploration University Hospital CHU G. Montpied INRA UNH CRNH Auvergne Clermont Auvergne University Clermont‐Ferrand France; ^3^ Faculty of Sports Science Ningbo University Ningbo China; ^4^ School of Health and Life Sciences Institute for Clinical Exercise & Health Science University of the West of Scotland South Lanarkshire UK; ^5^ Department of Biomedical Engineering University of Strathclyde Glasgow UK; ^6^ Department of Teaching Staff Training Faculty of Physical Education and Sports National University of Physical Education and Sports from Bucharest Bucharest Romania; ^7^ LaPSCo Catech Université Clermont Auvergne CNRS Clermont‐Ferrand France; ^8^ Health and Physical Education Program Indiana University of Pennsylvania Bloomington IN USA; ^9^ Laboratory of Metabolic Adaptations to Exercise under Physiological and Pathological conditions (AME2P) CRNH Auvergne Université Clermont Auvergne Clermont‐Ferrand France; ^10^ Department of Exercise Physiology University of Isfahan Isfahan Iran; ^11^ Department of Sport, Physical Education and Health Centre for Health and Exercise Science Research Hong Kong Baptist University Kowloon Tong China; ^12^ Biostatistics Unit DRCI University Hospital of Clermont‐Ferrand CHU Clermont‐Ferrand Clermont‐Ferrand France; ^13^ Preventive and Occupational Medicine University Hospital of Clermont‐Ferrand CHU Clermont‐Ferrand Clermont‐Ferrand France; ^14^ Physiological and Psychosocial Stress LaPSCo CNRS Preventive and Occupational Medicine WittyFit University Hospital of Clermont‐Ferrand CHU Clermont‐Ferrand Université Clermont Auvergne Clermont‐Ferrand France

**Keywords:** health, pandemic, vulnerable population

## Abstract

**Background:**

This paper is a follow‐up study continuing the COVISTRESS network previous research regarding health‐related determinants.

**Objective:**

The aim was to identify the main consequences of COVID‐19 lockdown on *Body Mass* *Index* and *Perceived Fragility*, related to *Physical Activity (PA),* for different categories of populations, worldwide.

**Design:**

The study design included an online survey, during the first wave of COVID‐19 lockdown, across different world regions.

**Setting and participants:**

The research was carried out on 10 121 participants from 67 countries. The recruitment of participants was achieved using snowball sampling techniques via social networks, with no exclusion criteria other than social media access.

**Main outcome measures:**

*Body Mass* *Index*, *Physical Activity*, *Perceived Fragility and risk of getting infected* items were analysed. SPSS software, v20, was used. Significance was set at *P* < .05.

**Results:**

*Body Mass* *Index* significantly increased during lockdown. For youth and young adults (18‐35 years), *PA* decreased by 31.25%, for adults (36‐65 years) by 26.05% and for the elderly (over 65 years) by 30.27%. There was a high level of *Perceived Fragility and risk of getting infected* for female participants and the elderly. Correlations between *BMI*, *Perceived Fragility* and *PA* were identified.

**Discussion and Conclusions:**

The research results extend and confirm evidence that the elderly are more likely to be at risk, by experiencing weight gain, physical inactivity and enhanced Perceived Fragility. As a consequence, populations need to counteract the constraints imposed by the lockdown by being physically active.

## 1 INTRODUCTION

In previous years, global pandemics have occurred, including COVID‐19, with severe consequences for world populations.^1^, ^2^ The COVID‐19 outbreak effects could be divided into two main areas of analysis. The first regards medical measures, which include identifying the genome sequence characteristics of the virus, finding adequate treatment for those infected, new research on discovering new diagnosis tools and COVID‐19 vaccine development strategies.^3^, ^4^ The second aspect is connected to the worldwide lockdown imposed by authorities in order to break the chain of infection and prevent further spread of the virus.^5^, ^6^, ^7^ Although lockdown constraints were different among countries and not simultaneously implemented, some regulations were mandatory worldwide: home confinement, closing down cultural and social events, teleworking, online schooling, restricted displacements, social distancing or restricted Physical Activity.^8^ These safety measures represented at the same time health‐related risk factors for *Body Mass* *Index* (*BMI*), *Physical Activity* (*PA*) and *Perceived Fragility and risk of getting* *infected*.

Evidence indicates that increased *BMI* is highly related to developing severe complications of COVID‐19.^9^ Authors documented a U‐shaped infection rate among overweight and underweight elderly,^10^ revealing that obesity is also implicated in impaired immune responses.^11^ It was emphasized that when examining the degree of association between higher *BMI* and the need of hospitalization, obesity was acknowledged as predicting a poor clinical prognosis in patients with COVID‐19.^12^, ^13^ Both obesity and underweight are proven to be important health‐related risk factors for various pathologies, including COVID‐19.^9^, ^14^ Research established causal mechanisms between overweight/obesity and the risk of developing severe symptoms of COVID‐19 in all age groups, especially in the elderly.^15^ Authors developed a risk prediction model for hospital admission and death rate from coronavirus in large cohort studies, including variables such as age, Body Mass Index, ethnicity, deprivation and a range of comorbidities.^16^

Studies examined the global changes in daily *PA*, revealing that the global trend emphasizes a drastic decline in *PA* levels across different countries and ages during the confinement period.^17^ This evidence needs formalization of *PA* guidelines comprising main recommendations in the areas of exercise and *PA*.^18^ Even if the lockdown was expected to protect against the virus, vulnerable people, namely the elderly and those with chronic medical conditions, were impacted by sedentary behaviour.^19^, ^20^ The evidence‐based consequences for the elderly during this pandemic highlight the importance of their perceived frailty, resulting from multiple comorbidities.^21^ Still, authors like Spiegelhalter (2020)^22^ discussed the slight difference between the normal risk of death and the COVID‐19 similar rate in England and Wales; before the pandemic, the fatality risk doubled every seven years of extra age, while in 2020 during a 16‐week pandemic period this risk doubled for every 5 to 6 years of extra age.

According to UN,^23^ there are more than 700 million people aged over 65 years worldwide, considered to be at higher risk for severe prognosis concerning COVID‐19,^24^ unless muscle function, cardiovascular fitness, immune function and overall well‐being ^25^, ^26^ are preserved. A wide understanding of fragility in the elderly requires a holistic vision including multiple causes: biological, psychological and social aspects, which render seniors more vulnerable to chronic illnesses, emotional stress or limited activities of daily living.^27^ Moreover, increased loneliness and reduced family interactions, along with *PA* restriction, are likely to produce health issues^28^, ^29^ that could be explained by alterations in the adaptive and innate immune systems.^30^

Regarding the meanings of the term ‘fragility’ from a biological perspective, meta‐analysis also addresses this concept as an individual trait greatly influenced by diverse physical and psychological stressors along with the diminished health system capacity to provide medical care.^31^ Fragility was described as a myriad of conditions such as decreased physiological reserve, emaciation or overweight, susceptibility to injuries and diminished stress resilience.^32^, ^33^ Moreover, specialists emphasize that the fragility syndrome is a more severe health status related to the simultaneous presence of two or more comorbidities.^34^, ^35^ Authors like Mello et al (2014)^36^ correlate the fragility syndrome with different characteristics, namely age, female gender, extreme BMI values, depressive symptoms and self‐evaluation health risks.

In the context of the COVID‐19 pandemic, the multidimensional fragility concept reveals to be even more relevant due to the increased threats for the individual health and a hindered stress response.^37^ Thus, feeling insecure and being worried about risk of infection are both consequences of a fragility status.

Fragility is also conceptualized by global entities like OECD as a complex political, economic and societal reality, requiring a distinct policy agenda and being largely influenced in the last year by the COVID‐19 pandemic. This reminded of the great disparities among countries in terms of health‐care systems.^38^

The aim of this study was to identify the main consequences of the COVID‐19 lockdown on *BMI* and *Perceived Fragility*, as relevant aspects related to *PA* as part of an active lifestyle. Conceived as a cross‐cultural study, this paper addressed different age, gender and weight categories, providing large‐scale information originating from numerous countries worldwide. It was not the intention of the authors to delineate specific national characteristics as to how the pandemic acted upon the individuals’ health outcomes. The research questions were the following: Were the COVID‐19 lockdown consequences equally distributed across different categories of individuals? Which of these categories were impacted the most by the restrictions linked to physical activities?

## 2 METHODS

### 2.1 Sample

This study involved 10 121 participants originating from 67 countries worldwide, which were analysed as a whole. They were asked to complete an online questionnaire linked to the COVID‐19 consequences on *BMI, PA and Perceived fragility and risk of getting infected*. The inclusion criteria for participants were the ability to complete this open‐access tool available at covistress.org.^39^ The recruitment of participants was achieved by self‐selection, using snowball sampling techniques via social media invitations (Facebook, Twitter, LinkedIn), where the aim, procedure and consent for participation were explained. No exclusion criteria were applied. All data were anonymized. The participants willingly gave their informed consent once they accessed the survey, having the possibility to withdraw at any time and/or partially complete the items, without giving reasons. Ethical approval for the research protocol was granted by the Research Ethics Commission of a University Hospital in Europe (Nos Ref.: 2020/CE 06, March 2020), the procedures applied complying with the Declaration of Helsinki. Each National representative institution provided Ethical approval.

### 2.2 Procedure

Our study encompassed the first COVID‐19 lockdown (March–May 2020), when severe regulations were applied in most countries experiencing different timings and durations for the lockdown. Data were collected from March to June 2020, allowing all participants to undergo COVID‐19 constraints. This research was a joint effort of universities, university hospitals and scientific research centres from France, Australia, Austria, Canada, Chile, China, Denmark, Indonesia, Italy, Iran, Norway, Portugal, Tunisia, Taiwan, Scotland, Switzerland, Romania and United States, all being part of an international research team. This COVISTRESS network (http://covistress.org/contacts.html) created an assessment tool intended to investigate the lifestyle and stress characteristics experienced by populations worldwide during the COVID‐19 pandemic. Concretely, a questionnaire was disseminated in different national languages, by means of REDCap^®^ application (Research Electronic Data Capture) specifically designed to create and manage online surveys.^40^, ^41^ The COVISTRESS questionnaire included 45 items divided into different sections: sociodemographic, epidemiological context, stress and worries, occupation and professions, parenthood and family, isolation and impact of coronavirus and health coverage.^42^ These items focused on collecting general information available worldwide with a common understanding of the aspects addressed, without making references to different countries. The completion of the questionnaire was estimated at 20 minutes. Within the current study, specific items were selected from the COVISTRESS questionnaire, as a stand‐alone approach, including the items relating to *BMI*, *PA* and *Perceived fragility and risk of getting infected*. The first two items referred to the prior pandemic period and to the first lockdown. Unlike BMI and PA, the *Perceived fragility and risk of getting infected* item made reference only to the lockdown period. Therefore, an accurate assessment of the situation at that time became possible. In order to identify the most vulnerable populations by age, the participants were divided into three categories: youth and young adults aged between 18 and 35 years old, adults aged between 36 and 65 years old and elderly aged over 65 years old. Depending on their *BMI*, the participants of most interest were the underweight and the overweight individuals. For the *PA* item, participants were requested to mention the number of hours of *PA* performed per week, before and during the isolation. For *Perceived Fragility*, the participants had to choose the intensity level of this measured outcome, from a 0 to 100 mm scale (Visual Analogue Scale). This scale has been used by different authors^43^, ^44^ to assess perceived stress, mental and physical fatigue. The task for the participants was to establish their level of *Perceived Fragility and risk of getting infected* on a horizontal line of 100 mm, ranging from very low (0), to very high (100).^45^

### 2.3 Statistics

Data analysis was performed using SPSS software, v20.^46^ The normality of the data distribution was ascertained using Skewness and Kurtosis coefficients. Skewness coefficient in absolute values was <1^47^; the symmetry (Skewness) and the flatness (Kurtosis) values were within normal limits,^48^ not exceeding a value of 3 for Skewness and 8 for Kurtosis. *t* tests were applied in order to determine the significant differences between prior and during sets of data. Significance was set at *P* < .05. According to Cohen, if d = 0.8 the effect size is strong, if d = 0.5, the effect size is medium, while for d = 0.2, the effect size is small.^49^ For the homogeneity of the variances, Levene's test was used.^50^ When the homogeneity condition was not met, an analysis of variance (ANOVA) was applied. Given the multiple comparisons performed on measured outcomes and the need to control the type 1 errors in null hypothesis, the powerful Hochberg test was applied.^51^ Correlational analysis was applied using either Spearman's or Pearson's coefficients. The correlations were considered to be very strong (*r* > .8), moderate (*r* = .6‐.8), fair (*r* = .3‐.6) and poor (*r* = .1‐.3).^52^

## 3 RESULTS

### 3.1 Socio‐demographic analysis

Out of the 10 121 participants who accessed the COVISTRESS questionnaire, 8124 totally or partially responded to the selected items. From the whole group, 5603 were female participants, with a mean age of 41.42 ± 12.97 years old, 2498 were male participants, with a mean age of 44.38 ± 14.28 years old, whereas 23 participants did not mention their sex. The subjects were divided into three age groups, namely 2759 youth and young adults aged between 18 and 35 years old, 4924 adults aged between 36 and 65 years old, 387 elderly aged over 65 years old, whereas 54 participants did not mention their age. All selected outcome measures had a normal distribution for the whole group by applying the Skewness and Kurtosis coefficients.

### 3.2 Body Mass Index analysis

Mean *BMI* value before isolation was 24.71 ± 5.04 kg/m² and increased significantly from a statistical standpoint during isolation to 24.78 ± 5.03 kg/m². *t* test value was t(7099) = 5.64, at *P* < .001, with a very small effect size (d = 0.01). The whole group of subjects fell into the healthy weight category. In females, mean *BMI* value before isolation was 24.32 ± 5.27 kg/m² (Skewness 1.60 (std. error 0.04) and Kurtosis 4.10 (std. error 0.07)) and increased significantly during isolation to 24.39 ± 5.26 kg/m² (Skewness 1.58 (std. error 0.04) and Kurtosis 3.99 (std. error 0.07)). *t* test value was t(4840) = 6.29, at *P* < .001, with a small effect size (d = 0.01). The female participants corresponded to the healthy weight category. For male participants, mean *BMI* value before isolation was 25.54 ± 4.39 kg/m² (Skewness 1.42 (std. error 0.05) and Kurtosis 4.82 (std. error 0.10)) and increased significantly during isolation to 25.60 ± 4.38 kg/m² (Skewness 1.40 (std. error 0.05) and Kurtosis 4.83 (std. error 0.10)). *t* test value was t(2223) = 2.1, at *P* = .04, with a very small effect size (d = 0.01). Both mean values expressed a slightly overweight category for the male participants. From the total number of participants completing this item, 457 were underweight (below 18.5 kg/m²), 492 were overweight and obese (25‐29.9 kg/m²; 30‐39.9 kg/m²), 6151 were healthy weight (18.5‐24.9 kg/m²). For the underweight, the mean *BMI* value before isolation was 18.06 ± 0.71 kg/m², (Skewness −0.86 (std. error 0.11) and Kurtosis 0.09 (std. error 0.23)) and increased significantly from a statistical standpoint during the isolation to 18.16 ± 0.87 kg/m², (Skewness −0.47 (std. error 0.11) and Kurtosis 0.62 (std. error 0.23)). *t* test value was t(456) = 3.88, at *P* < .001, with a very small effect size (d = 0.13). For the obese subjects, the mean *BMI* value before isolation was 36.78 ± 3.46 kg/m², (Skewness 0.99 (std. error 0.11), Kurtosis 0.45 (std. error 0.22)) and decreased significantly from a statistical viewpoint during isolation to 36.66 ± 3.60 kg/m², (Skewness 0.78 (std. error 0.11) and Kurtosis 0.51 (std. error 0.22)). *t* test value was t(491) = 2.14, at *P* = .033, with a very small effect size (d = 0.03). Both mean values referred to class II obesity. It is a common fact that obesity status most frequently leads to severe consequences for health outcomes. In general, the effect size, either small or very small, underlines the reduced differences between means. These differences were not likely to influence changes in health outcomes in the short term, during the first lockdown, although the value itself was highly concerning.

### 3.3 Physical Activity analysis

Regarding the number of hours of *PA* before isolation across three age groups, the data exhibited normal distribution. Despite this, the conditions of homogeneity of variances were not met, Levene's test indicated F(2,7198) = 44.753, *P* < .001, and as a result, the ANOVA test was applied. It was demonstrated that the subjects from the three age groups showed significantly different results [F(2,7198) = 53.24, *P* < .001] for *PA* before isolation, the effect size being small (η²*P* = .015). Hochberg test showed significant differences among the age groups (alpha=0.05). The effect size was small (d = 0.07) between 18‐35 years and 36‐65 years; over medium (d = 0.60) between 36‐65 years and over 65 years; and under medium (d = 0.44) between 18‐35 years and over 65 years. In terms of *PA* during isolation across three age groups, the data exhibited normal distribution. Despite this, the condition of homogeneity of variances was not met, with Levene's test indicating F(2,7176) = 14.71, *P* < .001, so the ANOVA test was applied. It was shown that the subjects from the age groups registered significantly different results [F(2,7198) = 30.06, <.001] for *PA* during isolation, the effect size being small (η²*P* = .008). Applying the Hochberg test, there were significant differences between the 18 and 35 years and over 65 years, as well as between 36 and 65 years and over 65 years, at alpha = 0.05. The effect size was almost medium (d = 0.43) for both 36‐65 years and over 65 years and between 18 and 35 years and over 65 years (d = 0.41) (Table 1).

**Table 1:** ANOVA test—*PA* per age groups

Source	Type III Sum of Squares	*df*	Mean Square	F	Sig.	Partial Eta Squared
*PA* before isolation						
Corrected Model	6361.7^a^	2	3180.83	53.25	<.001	0.015
Intercept	268 307.8	1	268 307.76	4491.38	<.001	0.384
Age group	6361.7	2	3180.83	53.25	<.001	0.015
Error	429 996.6	7198	59.74			
Total	1 015 668.0	7201				
Corrected Total	436 358.2	7200				
*PA* during isolation						
Corrected Model	2419.9^a^	2	1209.96	30.06	<.001	0.008
Intercept	132 808.8	1	132 808.82	3299.45	<.001	0.315
Age group	2419.9	2	1209.96	30.06	<.001	0.008
Error	288 846.9	7176	40.25			
Total	589 679.0	7179				
Corrected Total	291 266.8	7178				

^a^ *R* Squared = 0.015 (adjusted *R*‐squared = 0.014)–regression analysis.

### 3.4 Analysis for Perceived Fragility and risk of getting infected

Statistics corresponding to the whole group for *Perceived Fragility and risk of getting infected* revealed a mean value of 34.94 ± 32.02 on a scale from 0 to 100, with 36.33 ± 32.65 for females (Skewness 0.58 (std. error 0.03) and Kurtosis −0.98 (std. error 0.07)) and 31.71 ± 30.33 for the male participants (Skewness 0.76 (std. error 0.05) and Kurtosis −0.65 (std. error 0.10)). *t* test value was t_adjusted_(4880.1) = 6.03, at *P* < .001, with a small effect size (d = 0.14). With respect to the age groups, the mean value for the 18‐35 years was 27.12 ± 29.69 (Skewness 1.06 (std. error 0.05) and Kurtosis −0.02 (std. error 0.10)), 37.54 ± 32.09 for the 36‐65 years group (Skewness 0.53 (std. error 0.04) and Kurtosis −1.02 (std. error 0.07)) and 57.77 ± 31.49 for the over 65 years group (Skewness −0.38 (std. error 0.13) and Kurtosis −1.07 (std. error 0.25)). ANOVA emphasized that the participants from the three age groups registered significant different results [F(2,7710) = 198.3, *P* < .001]. The effect size was under medium (η²*P* = .049). Hochberg test demonstrated significant differences among all three age groups. The effect size was small (d = 0.33) between 18‐35 years and 36‐65 years; over medium (d = 0.63) between 36‐65 years and over 65 years; large (d = 1.02) between 18‐35 years and over 65 years.

### 3.5 Correlations among BMI, PA and Perceived Fragility and risk of getting infected

For the whole group, there were statistically significant correlations: a small positive correlation between *BMI* before and *Perceived Fragility and risk of getting infected* (*r* = .25); a very small negative correlation between *BMI* before and *PA* before (*r* = −.08); a small positive correlation between *BMI* during isolation and *Perceived Fragility and risk of getting infected* (*r* = .25); a small negative correlation between *BMI* during and *PA* during (*r* = .07). For female participants, there were statistically significant correlations: a small positive correlation between *BMI* before isolation and *Perceived Fragility and risk of getting infe*cted (*r* = .26); a very small negative correlation between *BMI* before isolation and *PA* before isolation (*r* = −.13); a small positive correlation between *BMI* during and *Perceived Fragility and risk of getting infected* (*r* = .27); a very small negative correlation between *BMI* during and *PA* during isolation (*r *= −.12). For male participants, statistically significant correlations were also found: a small positive correlation was identified between *BMI* before isolation and *Perceived Fragility and risk of getting infe*cted (*r* = .24); also, a small negative correlation between *BMI* before isolation and *PA* before isolation (*r* = −.05) and a small positive correlation between *BMI* during isolation and *Perceived Fragility and risk of getting infected* (*r* = .23) were revealed. For the underweight group, statistically significant correlations were identified: a small negative correlation was identified between the *BMI* before isolation and *Perceived Fragility and risk of getting infected* (*r* = −.11). Statistically significant correlations were emphasized for the obese group as well: a small positive correlation between *BMI* before isolation and *Perceived Fragility and risk of getting infected* (*r* = .20); a small negative correlation between *BMI* before isolation and *PA* before isolation (*r* = −.10); a small positive correlation between *BMI* during isolation and *Perceived Fragility and risk of getting infected* (*r* = .22); a small negative correlation between *BMI* during isolation and *PA* during isolation (*r* = −.16).

With regard to the comparative analysis for underweight and obese in terms of *Age*, *Perceived Fragility* and *PA*, the age mean values were 36.10 years for underweight and 44.03 years for the obese; for fragility, the mean values were 28.63 for the underweight and 57.20 for the obese; for *PA* before isolation, the mean values were 9.71 hours/week for the underweight and 7.08 hours/week for the obese; for *PA* during isolation, the mean values were 6.60 hours/week for the underweight and 5.06 hours/week for the obese. *t* test (Table 2) revealed significant differences between underweight and obese, in terms of age [t_adjusted_(890.4) = 9.49, *P* < .001]. The effect size was over medium (d = 0.62). The same test emphasized significant differences between underweight and obese in terms of *Perceived Fragility and risk of getting infected* [t_adjusted_(889.7) = 13.3, *P* < .001]. The effect size was large (d = 0.89). The *Perceived Fragility and risk of getting infected* were more intense in obese, compared with underweight: 57.2 ± 34.08 vs 28.63 ± 30.16. *t* tests also revealed significant differences between underweight and obese in terms of *PA* before isolation [t_adjusted_(856.7) = 5.01, *P* < .001], with a small effect size (d = 0.33). Prior to isolation, obese participants exercised less, compared with underweight: 7.08 ± 7.05 vs 9.71 ± 8.71. Regarding the *PA* during isolation, there were significant differences between underweight and obese [t_adjusted_(853.9) = 3.8, *P* < .001], with a small effect size (d = 0.25). During the isolation, the obese participants exercised less, compared to underweight participants: 5.06 ± 5.44 vs 6.6 ± 6.74.

**Table 2:** Independent‐samples test values among all outcome measures for the weight categories

Outcomes	Levene's Test for Equality of Variances	*t* test for Equality of Means (underweight versus obese)							
	F	Sig.	t	*df*	Sig. (2‐tailed)	Mean Difference	Std. Error Difference	95% Confidence Interval of the Difference	
								Lower	Upper
Age	16.94	<.001	−9.55	941	<.001	−7.94	0.83	−9.57	−6.31
			−9.49	890.4	<.001	−7.94	0.84	−9.58	−6.29
*Perceived Fragility and risk of getting infected*	15.40	<.001	−13.2	892	<.001	−28.60	2.16	−32.8	−24.3
			−13.3	889.7	<.001	−28.60	2.15	−32.8	−24.36
*PA* before isolation	18.38	<.001	5.05	924	<.001	2.63	0.52	1.61	3.65
			5.01	856. 7	<.001	2.63	0.52	1.60	3.65
*PA* during isolation	11.97	.001	3.83	923	<.001	1.54	0.40	0.75	2.33
			3.80	853.9	<.001	1.54	0.41	0.74	2.33

## 4 DISCUSSION

This paper is a continuation of our previous research regarding COVID‐19 consequences on different health‐related components, as conceptualized by the COVISTRESS network.^53^ This study emphasized a thematic sequence of this methodology, as complementary information to similar data collected by this team.

### 4.1 BMI

Despite the reduced *BMI* differences, which would not influence optimal health status in general, the statistical tools nonetheless revealed that for the whole group this outcome slightly varied before and during isolation. In the long term, an ascending trend for BMI, predictable under the circumstances of a prolonged pandemic, might represent a serious health risk for the participants. Similar results were reported for an Italian population of all ages and genders, exploring body weight changes during the lockdown.^54^ Our findings showed that female participants exhibited healthy weight *BMI* values both before and during isolation, while the male participants were slightly overweight both prior and during the lockdown. Although *BMI* registered a very small increase during isolation for both categories, female participants remained in the healthy weight category, while male participants remained slightly overweight. Weight gain, often correlated to its contributing factors, analysed during the pandemic, was confirmed in multiple studies for both genders, all of them raising awareness about the importance of nutritional status for a healthy lifestyle.^55^, ^56^ Taking into consideration the extreme weight categories as being linked to risk of severe COVID‐19 complications, we noticed that 13.11% of the total sample fell into these vulnerable categories. Severe COVID‐19 patients had overweight or obesity syndromes, reported as an ‘independent risk factor’, because enhanced adiposity diminishes pulmonary function.^57^

### 4.2 Physical Activity

According to recent studies,^58^, ^59^, ^60^ although the World Health Organization established the minimum duration of *PA* per day for a healthy person, the COVID‐19 pandemic has drastically restricted PA in all age groups, in all countries.^61^ In this study, statistical results provided insights about *PA* patterns before and during the isolation for different age groups. In all three age categories, the number of hours per week decreased by 31.25% for youth and young adults, by 26.05% for the adults and by 30.27% for the elderly, during the lockdown. Also, the participants over 65 years old were the most active before isolation, followed by the youth and young adults. During the lockdown, the young people were the most deprived, followed by the elderly and the adults. Active behaviour decreased also in the Italian young population and in the French elderly, due to severe restrictions regarding physical daily living activities.^62^, ^63^

### 4.3 Perceived Fragility and Risk of getting infected

This study emphasized a high level of *Perceived Fragility and risk of getting infected* for female participants, compared to the surveyed male population, which is also confirmed by different statistics.^64^ The most striking differences regarding *Perceived fragility and risk of getting infected* were identified among the three age groups. *Perceived fragility and risk of getting infected* gradually increased along with the age category. Thus, the over 65‐year participants had the most significant fragility score, most probably associated with their health status and fear of getting infected. Given the elderly multiple comorbidities, perceived risk was more than twice than that of the young. That is why the restrictive measures are more relevant when elderly subjects are involved.^65^, ^66^ Seniors facing great deprivation of family contact and social life in general will be able to cope with this new reality by adhering to COVID‐19 safety measures.^56^

### 4.4 Correlations among measured outcomes

According to statistical correlations, it was demonstrated that *BMI* influenced *Perceived Fragility* and had a cause‐effect relationship with *PA* performed before and during the isolation. *BMI* relating to physical self positively influences self‐confidence and empowerment, so that the individual feels less fragile in coping with COVID‐19. Regarding the same correlation applied to female and male subject outcomes, there were similar cause‐effect relationships between *BMI*, *Perceived Fragility* and the amount of *PA*, both before and during the lockdown, with the exception of the correlation between the male *BMI* and *PA* during isolation. With respect to the above‐mentioned correlations, it was obvious that for obese subjects, *BMI* related to their *Perceived Fragility* before and during the lockdown. The predicted relationship between *BMI* and the amount of *PA* was emphasized, both before and during lockdown, revealing the well‐known positive effects of exercise on physical appearance. Similar results regarding the benefits of Physical Activity on losing weight in obese patients were found on a regular basis before the pandemic onset.^67^ The results acknowledge that obese populations are at a higher risk of feeling physically fragile and prone to develop severe COVID‐19 symptoms if becoming infected.

### 4.5 Limitations

The research team was not able to control the severity of the restrictions or the time differences across countries. Also, different criteria for lockdown metrics were not taken into consideration for the surveyed countries, nor the differences between their economic status.

The self‐selection procedure for participants did not meet the requirements of the typical sampling techniques, but the great number of participants compensated for this limitation. The number of respondents varied to a great extent by country, the data being analysed as a whole, without underlining country‐specific aspects.

The present questionnaire did not include information about the exercise intensity or the type of *PA* performed. In terms of *Perceived Fragility*, further specific assessment tools are likely to add complementary information about the fragility syndrome in the elderly.

## 5 CONCLUSIONS

This study examined the main consequences of the first lockdown on different outcomes related to Physical Activity as part of an active lifestyle. Based on the research questions, the findings highlighted that the elderly, females and obese participants are more impacted by the COVID‐19 restrictions, especially regarding *PA*.

The current results extend and confirm previous research concerning vulnerable populations that are more likely to be at risk, by experiencing weight variations or an enhanced Perceived Fragility. We can conclude that physical and health‐related components might be impacted in the long term, by the changes in the daily routines imposed by the pandemic.

Vulnerable populations are more susceptible to developing different health conditions, without adequate counteracting measures to alleviate the physical and psychological curfew constraints. Despite the lockdown, individuals need to maintain an active lifestyle in order to tackle the pandemic challenges and to develop more resilient mechanisms to COVID‐19 infections.

To sum up, the health‐related findings in this paper reveal the current and potential effects upon long‐term health status, mostly triggered by the prolonged psychological pandemic stressors and diminished Physical Activity. In this context, health‐care professionals and also educators should focus on preventing sedentary lifestyle and providing psychological counselling as necessary interventions to mitigate some of the COVID‐19 consequences.

## CONFLICT OF INTEREST

The authors declare that there is no conflict of interest.

## AUTHORS CONTRIBUTION

The authors contributed equally to this work. CU and AB contributed to conceptualization; CU, AB and UCU contributed to methodology; MD, AB and CU contributed to software design; FD, MC, NA and YG validated the study; CU, AB, UCU and BP made formal analysis; CU, AB, UCU, BP and JSB made investigation; FD, DT, NA and KK contributed to resources; KK, YG, KR, NA and BP involved in data curation; CU and AB contributed to writing—original draft preparation; UCU, JSB, MD and RB contributed to writing—review & editing; FD and RB made visualization; FD and MC made supervision; FD and MC involved in project administration, and FD acquired funding. All authors have read and agreed to the published version of the manuscript.

## Data Availability

The data that support the findings of this study are available from the corresponding author upon reasonable request.

## References

[1] Al Zobbi M , Alsinglawi B , Mubin O , Alnajjar F . Measurement method for evaluating the lockdown policies during the COVID‐19 pandemic. Int J Environ Res Public Health. 2020;17(15):5574. 10.3390/ijerph17155574 PMC7432619 32748822

[2] Weston S , Frieman MB . COVID‐19: knowns, unknowns, and questions. mSphere. 2020;5(2):e00203‐20. 10.1128/mSphere.00203-20 32188753 PMC7082143

[3] Jeyanathan M , Afkhami S , Smaill F , Miller MS , Lichty BD , Xing Z . Immunological considerations for COVID‐19 vaccine strategies. Nat Rev Immunol. 2020;20(10):615‐632. 10.1038/s41577-020-00434-6 32887954 PMC7472682

[4] Pascarella G , Strumia A , Piliego C , et al. COVID‐19 diagnosis and management: a comprehensive review. J Intern Med. 2020;288(2):192‐206. 10.1111/joim.13091 32348588 PMC7267177

[5] Zheng L , Miao M , Lim J , Li M , Nie S , Zhang X . Is lockdown bad for social anxiety in COVID‐19 regions?: A national study in The SOR perspective. Int J Environ Res Public Health. 2020;17(12):4561. 10.3390/ijerph17124561 PMC7344490 32599911

[6] Yuen KS , Ye ZW , Fung SY , Chan CP , Jin DY . SARS‐CoV‐2 and COVID‐19: the most important research questions. Cell Biosci. 2020;10(1):40. 10.1186/s13578-020-00404-4 PMC7074995 32190290

[7] World Health Organization . Coronavirus Disease (COVID‐19) situation reports . 2020. https://www.who.int/emergencies/diseases/novel‐coronavirus‐2019/situation‐reports. Accessed September 30, 2020

[8] Rubin GJ , Wessely S . The psychological effects of quarantining a city. BMJ. 2020;368:m313. 10.1136/bmj.m313 31992552

[9] Malik VS , Ravindra K , Attri SV , Bhadada SK , Singh M . Higher body mass index is an important risk factor in COVID‐19 patients: a systematic review and meta‐analysis. Environ Sci Pollut Res Int. 2020;27(33):42115‐42123. 10.1007/s11356-020-10132-4 32710359 PMC7380664

[10] Dobner J , Kaser S . Body mass index and the risk of infection ‐ from underweight to obesity. Clin Microbiol Infect. 2018;24(1):24‐28. 10.1016/j.cmi.2017.02.013 28232162

[11] Carbone F , La Rocca C , De Candia P , et al. Metabolic control of immune tolerance in health and autoimmunity. Semin Immunol. 2016;28(5):491‐504. 10.1016/j.smim.2016.09.006 27720234

[12] Peres KC , Riera R , Martimbianco ALC , Ward LS , Cunha LL . Body mass index and prognosis of COVID‐19 infection. A systematic review. Front Endocrinol (Lausanne). 2020;11:562. 10.3389/fendo.2020.00562 32922366 PMC7456965

[13] Hussain A , Mahawar K , Xia Z , Yang W , El‐Hasani S . Obesity and mortality of COVID‐19. Meta‐analysis. Obes Res Clin Pract. 2020;14(4):295‐300. 10.1016/j.orcp.2020.07.002 32660813 PMC7346803

[14] Kim TS , Roslin M , Wang JJ , et al. BMI as a risk factor for clinical outcomes in patients hospitalized with COVID‐19 in New York. Obesity. 2021;29(2):279‐284. 10.1002/oby.23076 33128848 PMC8191573

[15] Popkin BM , Du S , Green WD , et al. Individuals with obesity and COVID‐19: a global perspective on the epidemiology and biological relationships. Obes Rev. 2020;21(11):e13128. 10.1111/obr.13128 32845580 PMC7461480

[16] Clift AK , Coupland CAC , Keogh RH , et al. Living risk prediction algorithm (QCOVID) for risk of hospital admission and mortality from coronavirus 19 in adults: national derivation and validation cohort study. BMJ. 2020;371:m3731. 10.1136/bmj.m3731 33082154 PMC7574532

[17] Tison GH , Avram R , Kuhar P , et al. Worldwide effect of COVID‐19 on physical activity: a descriptive study. Ann Intern Med. 2020;173(9):767‐770. 10.7326/M20-2665 32598162 PMC7384265

[18] Guerrero MD , Vanderloo LM , Rhodes RE , Faulkner G , Moore SA , Tremblay MS . Canadian children's and youth's adherence to the 24‐h movement guidelines during the COVID‐19 pandemic: a decision tree analysis. J Sport Health Sci. 2020;9(4):313‐321. 10.1016/j.jshs.2020.06.005 32525098 PMC7276134

[19] Rodrıguez M , Crespo I , Olmedillas H . Exercising in times ofCOVID‐19: what do experts recommend doing within four walls? Rev Esp Cardiol (Engl Ed). 2020;73(7):527‐529. 32414660 10.1016/j.rec.2020.04.001 PMC7142674

[20] Ricci F , Izzicupo P , Moscucci F , et al. Recommendations for physical inactivity and sedentary behavior during the Coronavirus disease (COVID‐19) pandemic. Front Public Health. 2020;8:199. 10.3389/fpubh.2020.00199 32574294 PMC7235318

[21] Cesari M , Pérez‐Zepeda MU , Marzetti E . Frailty and multimorbidity. different ways of thinking about geriatrics CONTROVERSIES IN LONG‐TERM CARE. J Am Med Dir Assoc. 2017;18(4):361‐364. 28279606 10.1016/j.jamda.2016.12.086

[22] Spiegelhalter D . Use of “normal” risk to improve understanding of dangers of covid‐19. BMJ. 2020;370:m3259. 10.1136/bmj.m3259 32907857

[23] United Nations . World Population Ageing 2019: s Division DoEaSA; 2019. https://www.un.org/en/development/desa/population/publications/pdf/ageing/WorldPopulationAgeing2019‐Highlights.pdf. Accessed September 30, 2020

[24] Amatriain‐Fernandez S , Gronwald T , Murillo‐Rodriguez E , et al. Physical exercise potentials against viral diseases like COVID‐19 in the elderly. Front Med (Lausanne). 2020;7:379. 10.3389/fmed.2020.00379 32714938 PMC7351507

[25] Damiot A , Pinto AJ , Turner JE , Gualano B . Immunological implications of physical inactivity among older adults during the COVID‐19 pandemic. Gerontology. 2020;66(5):431‐438. 10.1159/000509216 32585674 PMC7362590

[26] Holmes EA , O'Connor RC , Perry VH , et al. Multidisciplinary research priorities for the COVID‐19 pandemic: a call for action for mental health science. Lancet Psychiatry. 2020;7(6):547‐560. 10.1016/S2215-0366(20)30168-1 32304649 PMC7159850

[27] dos Santos RC , Almeida JLS , de Paiva Menezes RM , et al. Fragility syndrome in the elderly, integrating knowledge about diagnostic methods. Qual Prim Care. 2017;25(2):4.

[28] Brooks SK , Webster RK , Smith LE . The psychological impact of quarantine and how to reduce it: rapid review of the evidence. Lancet. 2020;395:912‐920. 32112714 10.1016/S0140-6736(20)30460-8 PMC7158942

[29] Frasquilho D , Matos MG , Salonna F , et al. Mental health outcomes in times of economic recession: a systematic literature review. BMC Public Health. 2015;16:115. 10.1186/s12889-016-2720-y PMC4741013 26847554

[30] Pedreañez A , Mosquera‐Sulbaran J , Muñoz N . SARS‐CoV‐2 infection represents a high risk for the elderly: analysis of pathogenesis [published online ahead of print, 2021 Mar 22]. Arch Virol. 2021;1‐10. 10.1007/s00705-021-05042-w PMC7982908 33751241

[31] Diaconu K , Falconer J , Vidal N , et al. Understanding fragility: implications for global health research and practice. Health Policy Plan. 2020;35(2):235‐243. 10.1093/heapol/czz142 31821487 PMC7050687

[32] McDermid RC , Stelfox HT , Bagshaw SM . Frailty in the critically ill: a novel concept. Crit Care. 2011;15(1):301. 10.1186/cc9297 21345259 PMC3222010

[33] Schoon Y , Bongers K , Van Kempen J , Melis R , Olde Rikkert M . Gait speed as a test for monitoring frailty in community‐dwelling older people has the highest diagnostic value compared to step length and chair rise time. Eur J Phys Rehabil Med. 2014;50(6):693‐701. 25077426

[34] Fried LP , Tangen CM , Walston J , et al. Cardiovascular Health Study Collaborative Research Group. Frailty in older adults: evidence for a phenotype. J Gerontol A Biol Sci Med Sci. 2001;56(3):M146‐M156. 11253156 10.1093/gerona/56.3.m146

[35] Mello JLC , Souza DMT , Chacara RAL , et al. Aging, fragility and palliative care: challenges of an emerging context. Hospice Palliat Med Int J. 2018;2(1):4. 10.15406/hpmij.2018.02.00057

[36] de Carvalho Mello A , Engstrom EM , Alves LC . Health‐related and socio‐demographic factors associated with frailty in the elderly: a systematic literature review. Cad Saúde Pública. 2014;30(6):1143‐1168. 10.1590/0102-311X00148213 25099040

[37] Bishop NA , Lu T , Yankner BA . Neural mechanisms of ageing and cognitive decline. Nature. 2010;464(7288):529‐535 20336135 10.1038/nature08983 PMC2927852

[38] OECD States of Fragility 2020 . 2020.

[39] Covistress Network . Covistress Questionnaire. http://covistress.org/index‐en.html. Accessed March 10, 2021.

[40] University College London . REDCap (Research Data Collection Service). UCL. https://www.ucl.ac.uk/isd/it‐for‐slms/redcap‐research‐data‐collection‐service. Accessed April 1, 2021.

[41] Patridge EF , Bardyn TP . Research Electronic Data Capture (REDCap). J Med Libr Assoc. 2018;106(1):142‐144. 10.5195/jmla.2018.319

[42] University of Clermont Auvergne . COVID‐19: UN VIRUS, DES IMPACTS. In: UCA , editor. Le journal de la Recherche de l’Université Clermont Auvergne no 8 ed: UCA; 2020.

[43] Lesage FX , Berjot S . Validity of occupational stress assessment using a visual analogue scale. Occup Med (Lond). 2011;61(6):434‐436. 10.1093/occmed/kqr037 21505089

[44] Lesage FX , Berjot S , Deschamps F . Clinical stress assessment using a visual analogue scale. Occup Med (Lond). 2012;62(8):600‐605. 10.1093/occmed/kqs140 22965867

[45] Dutheil F , Trousselard M , Perrier C , et al. Urinary interleukin‐8 Is a biomarker of stress in emergency physicians, especially with advancing age ‐ The JOBSTRESS* Randomized Trial. PLoS One. 2013;8(8):71658. 10.1371/journal.pone.0071658 PMC3747272 23977105

[46] IBM SPSS Statistics . 2020. https://www.ibm.com/analytics/spss‐statistics‐software. Accessed April 1, 2021.

[47] Morgan GA , Barrett KC , Leech NL , Gloeckner GW . chap 3 ‐ Measurement and descriptive statistics. IBM SPSS for Introductory Statistics: Use and Interpretation. 6th ed. New York: Routledge; 2019:47‐63.

[48] Kline R . Principles and Practice of Structural Equation Modeling, 3rd edn. New York: Guilford Press; 2011.

[49] Popa M . Statistica pentru psihologie: teorie si aplicatii SPSS (Statistics for Psychology: Theory and SPSS Applications), 2nd edn. Iasi: Polirom; 2008.

[50] Derrick B , Ruck A , Toher D , White P . Tests for equality of variances between two samples which contain both paired observations and independent observations. J Appl Quant Methods. 2018;13:36‐47.

[51] Benjamini Y , Hochberg Y . Controlling the false discovery rate: a practical and powerful approach to multiple testing. J R Stat Soc Series B Methodol. 1995;57:289‐300.

[52] Akoglu H . User's guide to correlation coefficients. Turk J Emerg Med. 2018;18(3):91‐93. 10.1016/j.tjem.2018.08.001 30191186 PMC6107969

[53] Ugbolue U , Duclos M , Urzeala C , et al. An assessment of the novel COVISTRESS questionnaire: COVID‐19 impact on physical activity, sedentary action and psychological emotion. J Clin Med. 2020;9(10):3352. 10.3390/jcm9103352 PMC7603364 33086648

[54] Di Renzo L , Gualtieri P , Pivari F , et al. Eating habits and lifestyle changes during COVID‐19 lockdown: an Italian survey. J Transl Med. 2020;18(1):229. 10.1186/s12967-020-02399-5 32513197 PMC7278251

[55] Đogaš Z , Lušić Kalcina L , Pavlinac Dodig I , et al. The effect of COVID‐19 lockdown on lifestyle and mood in Croatian general population: a cross‐sectional study. Croat Med J. 2020;61(4):309‐318. 10.3325/cmj.2020.61.309 32881428 PMC7480750

[56] Sun Z , Yang B , Zhang R , Cheng X . Influencing factors of understanding COVID‐19 risks and coping behaviors among the elderly population. Int J Environ Res Public Health. 2020;17(16):5889. 10.3390/ijerph17165889 PMC7460086 32823740

[57] Jose RJ , Manuel A . Does Coronavirus disease 2019 disprove the obesity paradox in acute respiratory distress syndrome? Obesity (Silver Spring). 2020;28(6):1007. 10.1002/oby.22835 32294322 PMC7262201

[58] Shahidi SH , Stewart Williams J , Hassani F . Physical activity during COVID‐19 quarantine. Acta Paediatr. 2020;109(10):2147‐2148. 10.1111/apa.15420 32557827 PMC7323361

[59] Lesser IA , Nienhuis CP . The impact of COVID‐19 on physical activity behavior and well‐being of Canadians. Int J Environ Res Public Health. 2020;17(11):3899. 10.3390/ijerph17113899 PMC7312579 32486380

[60] López‐Bueno R , Calatayud J , Andersen LL , et al. Immediate impact of the COVID‐19 confinement on physical activity levels in Spanish adults. Sustainability. 2020;12(14):5708. 10.3390/su12145708

[61] Karuc J , Sorić M , Radman I , Mišigoj‐Duraković M . Moderators of change in physical activity levels during restrictions due to COVID‐19 pandemic in young urban adults. Sustainability. 2020;12(16):6392. 10.3390/su12166392

[62] Goethals L , Barth N , Guyot J , Hupin D , Celarier T , Bongue B . Impact of home quarantine on physical activity among older adults living at home during the COVID‐19 pandemic: qualitative interview study. JMIR Aging. 2020;3(1):e19007. 10.2196/19007 32356777 PMC7207013

[63] Gallè F , Sabella EA , Ferracuti S , et al. Sedentary behaviors and physical activity of Italian undergraduate students during lockdown at the time of CoViD−19 pandemic. Int J Environ Res Public Health. 2020;17(17):6171. 10.3390/ijerph17176171 PMC7504707 32854414

[64] United Nations Women . UN Secretary‐General’s policy brief: the impact of COVID‐19 on women. United Nations Entity for Gender Equality and the Empowerment of Women (UN Women), United Nations Secretariat; 2020. p. 21.

[65] Okamura T , Ura C , Sugiyama M , et al. Defending community living for frail older people during the COVID‐19 pandemic. Psychogeriatrics. 2020;20(6):944‐945. 10.1111/psyg.12598 32803837 PMC7460990

[66] Armitage R , Nellums LB . COVID‐19 and the consequences of isolating the elderly. Lancet Public Health. 2020;5(5):e256. 10.1016/S2468-2667(20)30061-X 32199471 PMC7104160

[67] Jakicic JM , Davis KK . Obesity and physical activity. Psychiatr Clin North Am. 2011;34(4):829‐840. 10.1016/j.psc.2011.08.009 22098807

